# Supplementary material for: The effect of nurses’ individual and occupational characteristics and perceptions of hierarchical career plateau on their turnover intention: A cross-sectional study
Source: PLoS One. 2025 Apr 1;20(4):e0316895. doi: 10.1371/journal.pone.0316895 (PMC11960866; doi:10.1371/journal.pone.0316895)
Supplement: Attachment — Submitted filename: Response to Reviewers.docx [file pone.0316895.s003.docx]

**Manuscript ID:** PONE-D-24-39377

**Manuscript title:** The effect of nurses' individual and occupational characteristics and perceptions of hierarchical career plateau on their turnover intention: A cross-sectional study

| **Reviewers’ Comment** | **Response to Reviewers** | |  |
| --- | --- | --- | --- |
| **Reviewer1:** | Dear reviewer  Thank you for reviewing this study and providing valuable suggestions. | |  |
| **1.Comment:**  The first line of abstract should be start with the background of the study. It is directly started from the objective which seems inappropriate. | The first line of the abstract was started with the background of the study. The added sentence is shown below:  “Career plateau is associated with undesirable work outcomes such as low job performance, motivation, work and organizational commitment, increased counterproductive work behaviors, and turnover intentions. The aim of the present study was to examine the effect of nurses' personal and professional characteristics and their perceptions of hierarchical career plateau on their turnover intentions.” | Page 2  Line Number: 24-28 | |
| **2.Comment:**  The line starting from Data were collected…. Includes both the methodology and analysis which should be separate as in the next line it is written that multivariate linear regression analysis was used….. so the author can remove the “anlalyzed using linear regression” term in the previous line. | The data collected was removed from the sentence analyzed using linear regression. The sentence was modified as follows:  “Data were collected using a Nurse Information Form, the Career Plateau Survey and Turnover Intention Scale.” | Page 2  Line Number:34-35 | |
| **3.Comment:**  Independent variables should be mentioned in the abstract as the dependent variable is mentioned here. | Independent variables are indicated in the summary:  “Multivariate linear regression analysis was used to determine the effect of independent variables (individual and occupational characteristics and perceptions of hierarchical career plateau) on the dependent variable (turnover intention).” | Page 2  Line Number: 35-36 | |
| **4.Comment:**  In introduction part nothing is mentioned regarding the turnover intention. Firstly, the author should mention about what is turnover intention and then try to link it with the literature reviews**.** | Turnover intention has been defined:  “Turnover intention is defined as an employee’s reported willingness to leave the organization within a defined period of time [1], which includes a conscious, deliberate [2] and multi-stage process [3].” | Page 3  Line Number: 48-50;  Line Number:  52-53 | |
| **5.Comment:**  There is no information about the non-respondents or non-response item. The author should mention about the sample size after excluding the non-responses also in the study. Is all 464 sampled nurses responded to the form? | The following sentences have been added to the sample section:  “A "required to answer" sign was added to all questions in the questionnaire. Nurses participating in the study could not proceed to the next question or submit the form without responding the previous one. A total of 464 nurses who completed the questionnaire constituted the sample of the study. ” | Page 6  Line Number:  139-142 | |
| **6.Comment:**  Table 1 title should be Profile of the respondents | The title of Table 1 has been changed to “Profile of the respondents”. | Page 10  Line Number:  217 | |
| **7.Comment:**  In table 2, *p<0.001 description is not mentioned. It should be p<0.01 at 1% level of significance. | In Table 2, the significance level has been changed from "*p<0.001" to "*p<0.01". | Page 11  Line Number:  229. | |
| **8.Comment:**  The variables of table 3 should be described in a brief and proper way. | Table 3 has been revised. | Page 12  Line Number:  240. | |
| **9.Comment:**  Multivariate analysis is not clear as it is totally messed up and not indicates the concise clear result. Rewrite it. | Multivariate analysis results have been rewritten:  “Multivariate linear regression analysis was conducted to identify the independent variables associated with nurses' turnover intention (Table 4).  The model explained approximately 70.5% of the variance in the total turnover intention score. An increased hierarchical career plateau (β = 0.738; p < 0.001) and working in a public institution (β = 0.189; p < 0.001) were found to have a positive effect on turnover intention, while willingly choosing the nursing profession (β = -0.349; p < 0.001) had a preventive effect on turnover intention (Table 4).“ | Page 13  Line Number:  245-250. | |
| **10.Comment:**  Reformat the table 4. The result of linear regression model formatting is totally weird. Alignment and proper capitalization of words are necessary. | Table 4 has been revised | Page 13  Line Number:  251. | |
| **11.Comment:**  In the discussion part, the line starting with “In a study ……….” Is wrong. This study is conducted by the author that’s why it should be stated like “ In the present study, it was tried to examine………” and also write all the results and discussion part in past tense as this study was already completed. | The first sentence of the discussion has been changed to read as follows:  “In the present study, the effect of nurses' individual and occupational characteristics and their perceptions of hierarchical career plateau on turnover intention was examined.” | Page 13  Line Number:  255. | |
| **12.Comment:**  Many times “ In the study” phrase is used everywhere, try to rewrite the sentences with proper alignment and sentence reframing. | Some of the sentences beginning with "In a study" have been rewritten by removing "In the present study" from some sentences. | Page 14-20 | |
| **13.Comment:**  Write all the results and introduction or all over content in your own words or refer to standard paper language. This paper uses too much use AI tool such as Chatgpt. | Chatgpt was not used in this study. However, it has been reviewed in accordance with your comment. | All of the text | |
| **Comment:**  Is the manuscript presented in an intelligible fashion and written in standard English?  PLOS ONE does not copyedit accepted manuscripts, so the language in submitted articles must be clear, correct, and unambiguous. Any typographical or grammatical errors should be corrected at revision, so please note any specific errors here. | The article has been rewritten in English. | All of the text | |
| **Reviewer 2:** | Dear reviewer  Thank you for reviewing this study and providing valuable suggestions. | |  |
| **1.Comment:**  The manuscript is great and the data support the conclusions.  The statistical analysis has been performed appropriately and rigorously. | Thank you for reviewing this study. | All of the text | |
| **2.Comment:**  The authors have made all data underlying the findings in their manuscript fully available.  The manuscript is presented in an intelligible fashion and written in standard English. | Thank you for reviewing this study. |  | |
| **3.Comment:**  Overall, this manuscript is good, but several things need to be considered, such as the period of the reference sources used.I think it is better to use references within the last 5 years. | Some of the references from the last 5 years have been removed. | Page 21-29 | |
| **Reviewer 3:** | Dear reviewer  Thank you for reviewing this study and providing valuable suggestions. |  | |
| **1.Comment:**  The study provides valuable insights into the impact of career plateaus on nurses’ turnover intention.  Given the above, consider using thematic subheadings in the discussions to reflect the study’s results and enhance the readability of the discussion. | Thematic subheadings were used in the discussions. | Page 14  Line Number:  260, 280, 309, 384. | |
| **2.Comment:**  Again, consider expanding the discussion on how different hospital types influence turnover intention. | The discussion of how different types of hospitals affect turnover intentions was expanded. | Page 16  Line Number:  309-383. | |
| **3.Comment:**  Please ensure that all references are up-to-date and relevant to the study’s context, while you also review for some minor grammatical corrections entirely. | Some references not within the last 5 years have been removed. Some references not relevant to the context of the study have been removed. The article has been rewritten in English. | All of the text | |
| **4.Comment:**  Concerning formatting of the work, please ensure that the tables and work are well formatted to reduce the occurrence of blank spaces and tables spilling over to other pages. | Tables have been revised and edited. | Page 30-34 | |
| **Reviewer 4:** | Dear reviewer  Thank you for reviewing this study and providing valuable suggestions. |  | |
| **1.Comment:**  The research is conducted in a scientific and organized manner, contains the research methodological conditions, and is accepted for publication.I commend the effort made in the research and the organized work and achievement by the researchers of the research. | Thank you for reviewing this study. |  | |
| **Journal requirements:** | Thank you for reviewing this study and providing valuable suggestions. |  | |
| Kindly provide a summary of the conclusion. It is excessively lengthy. | Summary abbreviated. | Page 20  Line Number:  423-436 | |
| **1.Comment:**  Please ensure that your manuscript meets PLOS ONE's style requirements, including those for file naming. | Revised for PLOS ONE style. | All of the text | |
| **2.Comment:**  In this instance it seems there may be acceptable restrictions in place that prevent the public sharing of your minimal data. However, in line with our goal of ensuring long-term data availability to all interested researchers, PLOS’ Data Policy states that authors cannot be the sole named individuals responsible for ensuring data access (http://journals.plos.org/plosone/s/data-availability#loc-acceptable-data-sharing-methods).  Data requests to a non-author institutional point of contact, such as a data access or ethics committee, helps guarantee long term stability and availability of data. Providing interested researchers with a durable point of contact ensures data will be accessible even if an author changes email addresses, institutions, or becomes unavailable to answer requests.  Before we proceed with your manuscript, please also provide non-author contact information (phone/email/hyperlink) for a data access committee, ethics committee, or other institutional body to which data requests may be sent. If no institutional body is available to respond to requests for your minimal data, please consider if there any institutional representatives who did not collaborate in the study, and are not listed as authors on the manuscript, who would be able to hold the data and respond to external requests for data access? If so, please provide their contact information (i.e., email address). Please also provide details on how you will ensure persistent or long-term data storage and availability. | The SPSS file for the study data has been added to the appendices. In this way, researchers can freely access the data. | All of the text | |
